# Genetic complementation fosters evolvability in complex fitness landscapes

**DOI:** 10.1038/s41598-022-26588-y

**Published:** 2023-01-12

**Authors:** Ernesto Segredo-Otero, Rafael Sanjuán

**Affiliations:** Institute for Integrative Systems Biology (I2SysBio), Consejo Superior de Investigaciones Científicas-Universitat de València, C/ Catedrático Agustín Escardino 9, 46980 Paterna, València, Spain

**Keywords:** Computer modelling, Evolvability, Virology, Evolution, Genetics

## Abstract

The ability of natural selection to optimize traits depends on the topology of the genotype-fitness map (fitness landscape). Epistatic interactions produce rugged fitness landscapes, where adaptation is constrained by the presence of low-fitness intermediates. Here, we used simulations to explore how evolvability in rugged fitness landscapes is influenced by genetic complementation, a process whereby different sequence variants mutually compensate for their deleterious mutations. We designed our model inspired by viral populations, in which genetic variants are known to interact frequently through coinfection. Our simulations indicate that genetic complementation enables a more efficient exploration of rugged fitness landscapes. Although this benefit may be undermined by genetic parasites, its overall effect on evolvability remains positive in populations that exhibit strong relatedness between interacting sequences. Similar processes could operate in contexts other than viral coinfection, such as in the evolution of ploidy.

## Introduction

Evolvability is determined by the topology of the genotype-fitness map (fitness landscape), among other factors such as populations size, reproduction mode, and mutation rate. The topology of fitness landscapes depends on gene-by-gene interactions (epistasis)^1–4^. With no epistasis, gradual evolutionary optimization is straightforward because selection can operate on a succession of mutations, each conferring a certain fitness benefit independent of other mutations. In contrast, when traits are encoded by multiple genes exhibiting abundant epistasis, maladaptive allele combinations introduce low-fitness valleys between local fitness peaks. Such ruggedness represents a major obstacle to evolutionary optimization, since natural selection per se cannot favor intermediate, low-fitness states. Evidence from experimental evolution and mutant analysis studies suggests that epistasis is widespread and that, consequently, most fitness landscapes are rugged^5–8^. Epistasis among positions within a gene (conformational epistasis) may also play a key role in protein evolution^9^.

Evolutionary innovation often requires exploration of rugged fitness landscapes, since novel traits need not to be reached by gradual modification of pre-existing ones. Several explanations have been put forward to explain how the adaptation process takes place in epistatic fitness landscapes and, thus, how populations are able to jump from local maxima to higher fitness peaks. First, with sufficiently high mutation rates, certain combinations of favorable alleles could appear simultaneously, avoiding low-fitness intermediates^10^. However, high mutation rates are unlikely to supply complex adaptations in a single step, and would strongly increase mutational load, which in turn burdens adaptation. Second, episodes of random genetic drift may allow populations to traverse local fitness valleys and subsequently reach new fitness peaks under the action of natural selection^11^. Third, theoretical and experimental work has revealed that organisms exhibit certain features such as genetic redundancy, and other mutational tolerance mechanisms, that favor the accumulation of cryptic genetic variation in populations. These conditionally neutral variants can promote evolvability, as they may become beneficial upon changes in genetic background or environment^12,13^. It has also been shown that the very action of natural selection can promote evolvability by increasing mutational robustness^14^. Finally, mechanisms of phenotypic plasticity, stochastic gene expression, epigenetics and resiliency under adverse conditions (e.g. latency) might facilitate the evolution of novel traits in the long term^1^. However, the genetic and population-level determinants of evolvability remain to be investigated further.

Here, we examine whether complementation between different genetic variants can foster evolvability in rugged fitness landscapes. To this end, we simulated populations in which groups of sequences mutually compensate their genetic defects. We contextualized our hypothesis within the field of viral evolution. This allowed us to investigate the implications and limitations of the model in a specific type of biological system, although the model should in principle be applicable to other systems. There are at least two reasons why viruses should be particularly prone to genetic complementation. First, they typically exhibit extremely high levels of sequence diversity as a result of their high rates of spontaneous mutation^15–17^. Second, coinfections are relatively frequent^18–20^. Coinfection of a given cell with multiple viral particles of the same species can occur when there is a high local ratio of viral particles to susceptible cells (multiplicity of infection), but also when groups of viral infectious particles are jointly transmitted^21^. An example of the later process is provided by extracellular vesicles containing multiple viral particles^22^, which have been described in widely different types of viruses including hepatitis A virus^23^, enteroviruses^24^, rotaviruses^25^, noroviruses^25^, and marseilleviruses^26^. Baculovirus occlusion bodies constitute another example of collective viral spread in which transmission between insect hosts involves pools of nucleocapsids embedded in polyhedrin protein crystals^27^. Yet another example of collective viral spread is aggregation of infectious particles in the extracellular milieu, which has been described in different types of viruses including human immunodeficiency virus particles in semen^28^, and vesicular stomatitis virus particles in saliva^29^. The delivery of multiple genetic variants of a given virus to the same cell allows different viral genomes to share gene products in cooperative or antagonistic manners^30^.

Our simulations show that genetic complementation can increase evolvability by allowing low-fitness allele combinations to be maintained in the population. In this way, populations gain the ability to traverse fitness valleys and reach high-fitness peaks that are not directly accessible through a simple mutation-selection process. We also investigate the stability of this form of cooperation in the face of genetic parasites and find that, although parasites severely undermine population fitness and evolvability, this interference can be overcome in highly structured populations where interactions take place preferentially among genealogically related sequences.

## Methods

### Basic mutation-selection model

We developed a stochastic model in which haplotypes or sequences consist of a binary string of length *N*. Each string had a certain probability of being selected for the next generation depending on its fitness (selection), and each of the *N* positions had probability *α* of undergoing mutation by changing '0' to '1', or vice versa (mutation). The population was composed of *S* sequences (typically 10,000), and in each generation *S* new sequences were selected by drawing *S* times a sequence from the population. In each draw, a sequence *i* had a probability $P_{i} = f_{i} /\mathop \sum \limits_{j}^{S} f_{j}$ of being selected, where *f*_*i*_ is fitness. Thus, each sequence had a probability $1 - (1 - f_{i} /\mathop \sum \limits_{j}^{S} f_{j} ) ^{S}$ of contributing to the next generation. The fitness of each sequence was obtained from a predefined fitness landscape, which assigned a fitness value to each binary string.

**Figure 1:**
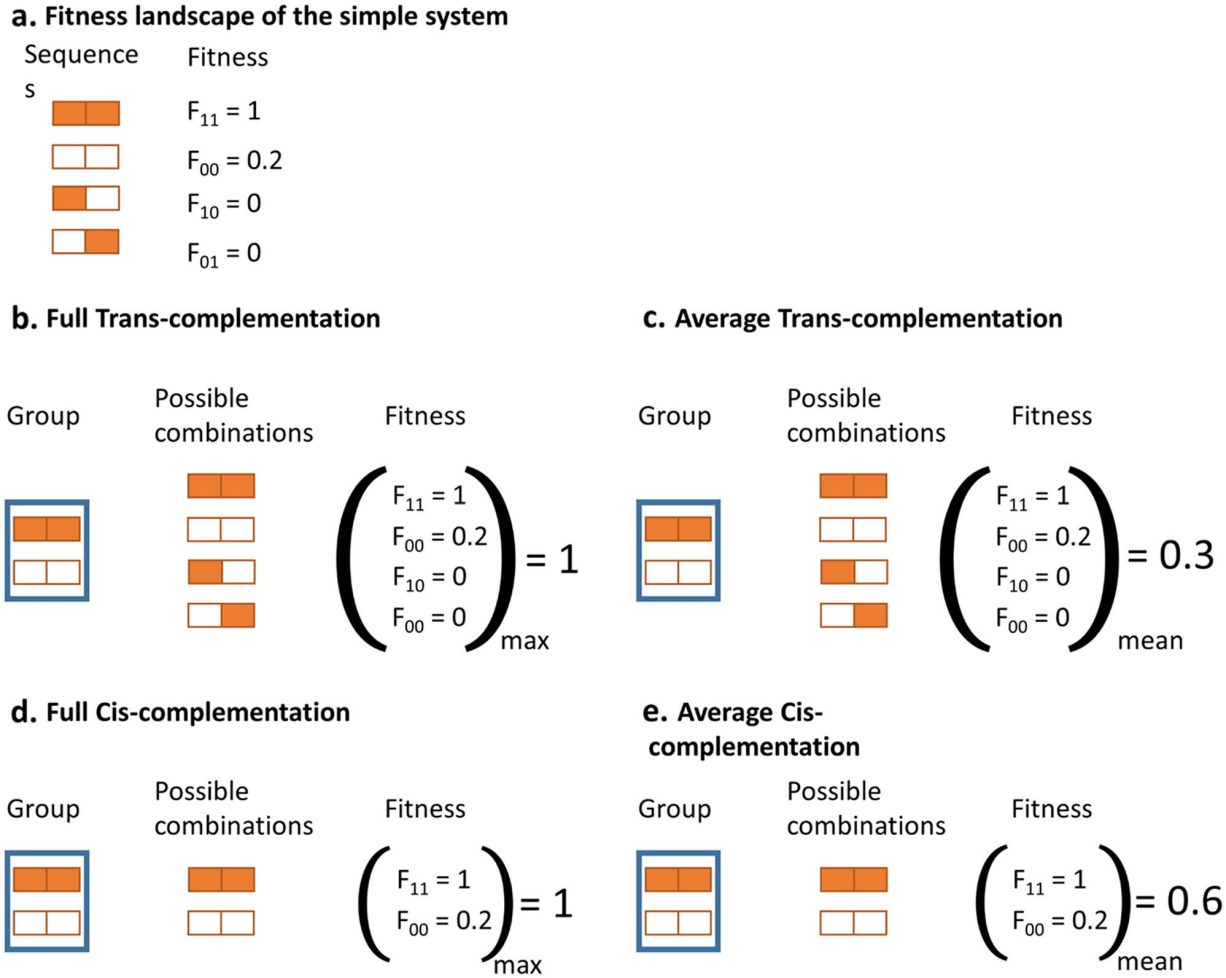
Definitions of genetic complementation. The simplest, two-locus two-allele system is shown. The two alleles are shown in orange and white. (**a**) Arbitrarily defined landscape that assigns a fitness value to each individual sequence. (**b**–**d**) Different rules for determining the fitness of pairs of complementing sequences. (**b**) Full trans-complementation, in which group fitness is determined by the fittest allele combination. (**c**) Average trans-complementation, in which group fitness equals the average fitness of all allele combinations. (**d**) Full cis-complementation, in which group fitness is determined by the fittest sequence. (**e**) Average cis-complementation, in which group fitness equals the average fitness of individual sequences.

The simplest scenario considered was a two-locus system with two alleles per locus. In this model, there were only four possible sequences: 0–0, 0–1, 1–0 and 1–1 (Fig. 1a). To introduce epistasis, we defined a landscape with a local maximum in the 0–0 sequence and a global maximum in the 1–1 sequence. These two peaks were separated by the low-fitness states 1–0 and 0–1. To generalize the model for multiple loci and epistasis, we used random *NK* models^31^. These fitness landscapes are built randomly, creating sequences of length *N* with *K* epistatic interactions per site. The fitness values of the sequences are calculated as a random contribution of each of the *N* positions, depending not only on its state (0 or 1), but also on the state of other *K* random positions. *K* = 0 produces a purely additive (non-epistatic) fitness landscape, while *K* = *N–*1 produces a random landscape with zero correlation between neighboring sequences (House of Cards). Increasing *K* increases the ruggedness of the landscape and, consequently, the number of local peaks.

### Genetic complementation

To allow for genetic complementation, we created groups of sequences and calculated a shared fitness value for each group. Specifically, the population of *S* sequences was structured in *S*/*m* of groups of size *m*. We used different rules to calculate the fitness value associated to groups. First, we considered classical genetic trans-complementation, whereby genes from different genomes share their gene products to generate a phenotype. For this, we obtained all possible allele combinations produced by the sequences in a group, and used the same predefined fitness landscape used in the basic selection model to calculate the fitness of each of these combinations. For example, in groups formed by sequences (1–1) and (0–0), there are four possible allele combinations: (1–1), (1–0), (0–1) and (0–0). Group fitness was calculated in two alternative ways: as the fitness of the fittest allele combination present in the group (Fig. 1b), or as the average fitness of all these allele combinations (Fig. 1c). The former represents a scenario in which a single copy of a gene is sufficient to recover the fittest phenotype (full trans-complementation), while the latter represents a system in which gene dosage determines the phenotype (average trans-complementation).

We also modeled a different type of complementation, in which the elements of a sequence did not represent different loci, but different positions within a locus (nucleotides, amino acids, cis-acting elements). We called this ‘cis-complementation’. Here, we no longer considered all possible combinations of alleles, but only each actual sequence present in a group (Fig. 1d–e). Conformational epistasis^9^ is an example of this situation, where a single gene must accumulate several mutations to produce a protein with a different phenotype. As in the previous case, the fitness of the group was calculated as the fitness of the fittest sequence (full cis-complementation, Fig. 1d), or as the average of all sequences within the group (average cis-complementation, Fig. 1e).

### Selection in the presence of complementation

When groups of complementing sequences were defined, we calculated the fitness of each sequence in a two-step manner. First, we computed the fitness of each group (*f*_*g*_) using the above-defined rules. The probability that a group contributed to the next generation, in a single draw, was $P_{g} = f_{g} /\mathop \sum \limits_{j}^{G} f_{j}$. Then, we obtained the fitness of each individual sequence within a group. In the absence of intra-group conflict, all group members had the same fitness, meaning that the probability of an individual *i* within group *g* of being selected in each draw was $P_{i|g} = \frac{1}{m}P_{g}$. In contrast, in the presence of genetic parasites, some sequences took more benefits from the group than others, thus behaving as social cheaters in a cooperative system. Specifically, the probability that individual *i* within group *g* was drawn became $P_{i|g} = P_{g} \beta_{i} /\mathop \sum \limits_{k}^{m} \beta_{k}$. In all simulations we used *β*_*i*_ = 1 for ‘normal’ (helper) sequences and *β*_*i*_ = 5 for genetic parasites. The simulations started with a population of helper sequences but, in each generation, helpers had a certain probability of mutating to parasites (*α*_*C*_). In viruses, genetic parasites often consist of genomes with large deletions^32,33^, called defective interfering particles, which are unable to successfully infect cells on their own or to complement deleterious genes from other viruses. To capture these features, we made the following assumptions: first, fitness was null for groups containing parasites only. Second, parasitic sequences contained a random number of deleted genes (starting by the first position, we deleted the first *I* genes, *I* being a uniformly distributed integer between 1 and *N*) such that, for these genes, the parasites were unable to complement deleterious alleles present in other sequences.

To obtain the average fitness effects of mutations through the evolution process, we calculated, in each generation, the fitness of each group before and after mutating (changing ‘0’ to ‘1’ or vice versa) individually each allele in each sequence of the population.

Simulations were implemented in MATLAB R2018. Scripts are available upon request.

## Results

### Genetic complementation promotes evolvability in a two-sequence, two-locus, two-allele model

**Figure 2:**
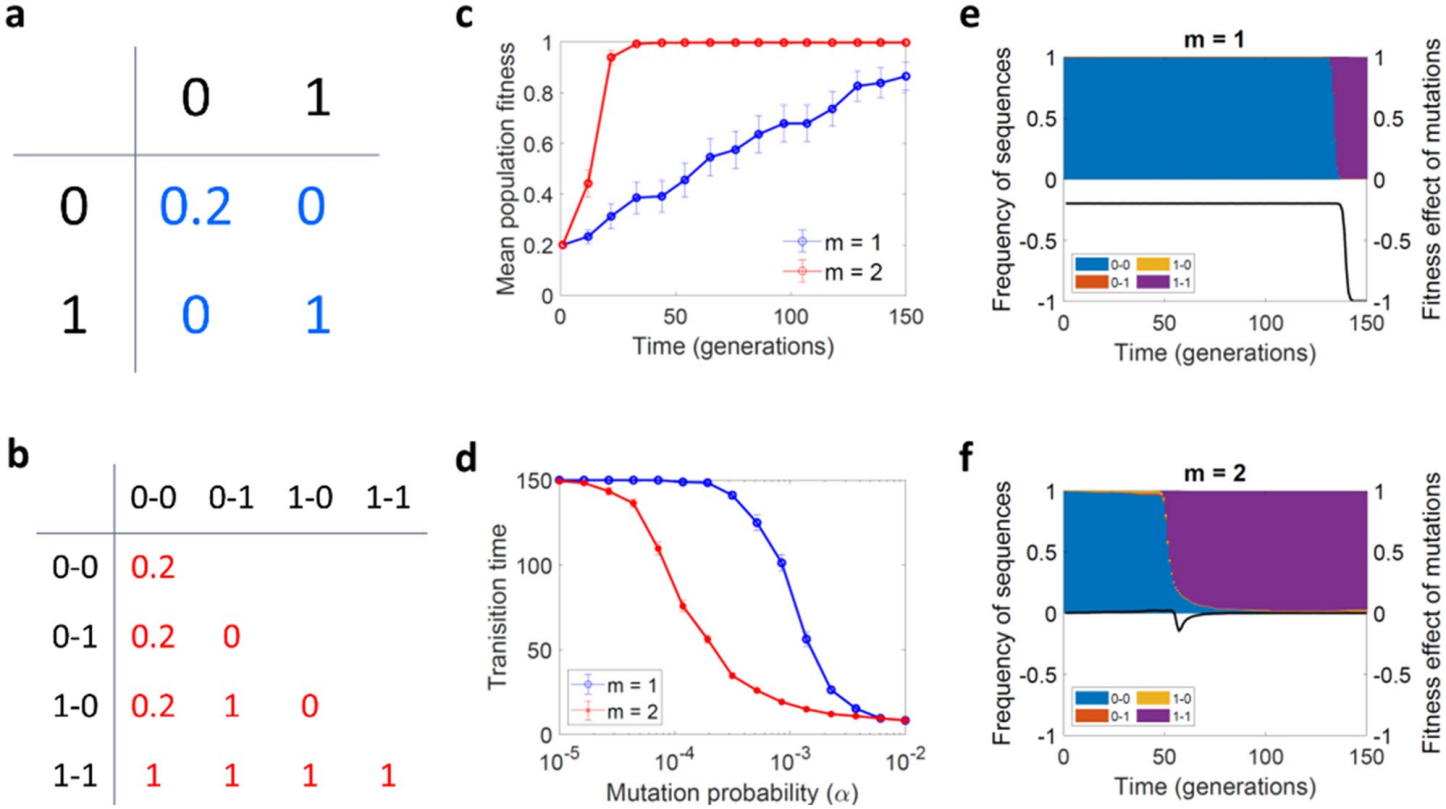
Genetic trans-complementation promotes evolvability in a two-sequence, two-locus, two-allele model. (**a**) Fitness values for each of the four possible sequences. (**b**) Fitness values for each of the 10 possible two-by-two interactions. (**c**) Evolution of mean population fitness in the presence (red) or absence (blue) of trans-complementation between pairs of sequences. (**d**) Average number of generations required for the population to reach a mean fitness higher than 0.95 as a function of the mutation rate, *α*. (**e**–**f**) Individual simulations showing the frequency of each sequence (0–0, 0–1, 1–0, and 1–1) and the average fitness effect of individual mutations (black line) as a function of time. *Parameters* *S* = 10,000, and in **c** and **e**–**f**, *α* = 0.001. In **c**–**d**, we show the average and SEM from 100 simulations.

We started our simulations with the simplest possible case, a two-locus, two-allele system with groups of two sequences (*m* = 2), which yields four possible individual sequences (0–0, 1–0, 0–1 and 1–1) and 10 different sequence pairs. This will be later generalized to multiple loci and larger groups. To investigate how evolutionary optimization takes place in the presence of deleterious intermediates, we assigned each of the four sequences the following fitness values: *f*_*11*_ = 1, *f*_*00*_ = 0.2 and *f*_*10*_ = *f*_*01*_ = 0 (Fig. 2a). The population initially consisted exclusively of 0–0 sequences, and we explored whether the 1–1 sequence was reached by a stochastic mutation-selection process. In the absence of interactions between different sequences, this could only occur via a double mutation transforming directly 0–0 into 1–1, since each mutation alone was lethal. To introduce complementation, we formed random pairs of individuals and allowed them to mutually compensate for their genetic defects (trans-complementation; Fig. 1b–c). We first assumed that the fitness of a pair was determined by the best allele combination present in each group (full trans-complementation; Fig. 2b). For instance, in pairs of 0–1 and 1–0 sequences both deleterious alleles would be rescued, allowing each sequence to reach a fitness value equal to *f*_*11*_. We found that this form of cooperation accelerated evolutionary optimization in this simple model, as evidenced by the fact that, in populations forming groups of size two (*m* = 2), the number of generations required for the population to reach the high-fitness sequence 1–1 was reduced (Fig. 2c–d). The 1–1 sequence was accessed faster with complementation because mutations in the founder sequence 0–0 tended to be neutral. This allowed the 0–1 and 1–0 sequences to reach a certain population frequency which, although small, was high enough to facilitate the emergence of the 1–1 sequence after an additional mutation. With complementation, neutrality was predominant throughout the evolution process, except during the transition time between the 0–0 to the 1–1 sequences. During this period, certain pairs were susceptible to mutation such as, for instance, groups formed by 1–1 and 0–1 sequences, in which back mutation of the 1–1 sequence to 0–1 was deleterious. In contrast, in the absence of complementation, the intermediate states 0–1 and 1–0 were strongly disfavored by selection, making the emergence of the 1–1 sequence less likely (Fig. 2e–f).

### Genetic parasites undermine complementation but can be avoided in highly structured populations

**Figure 3:**
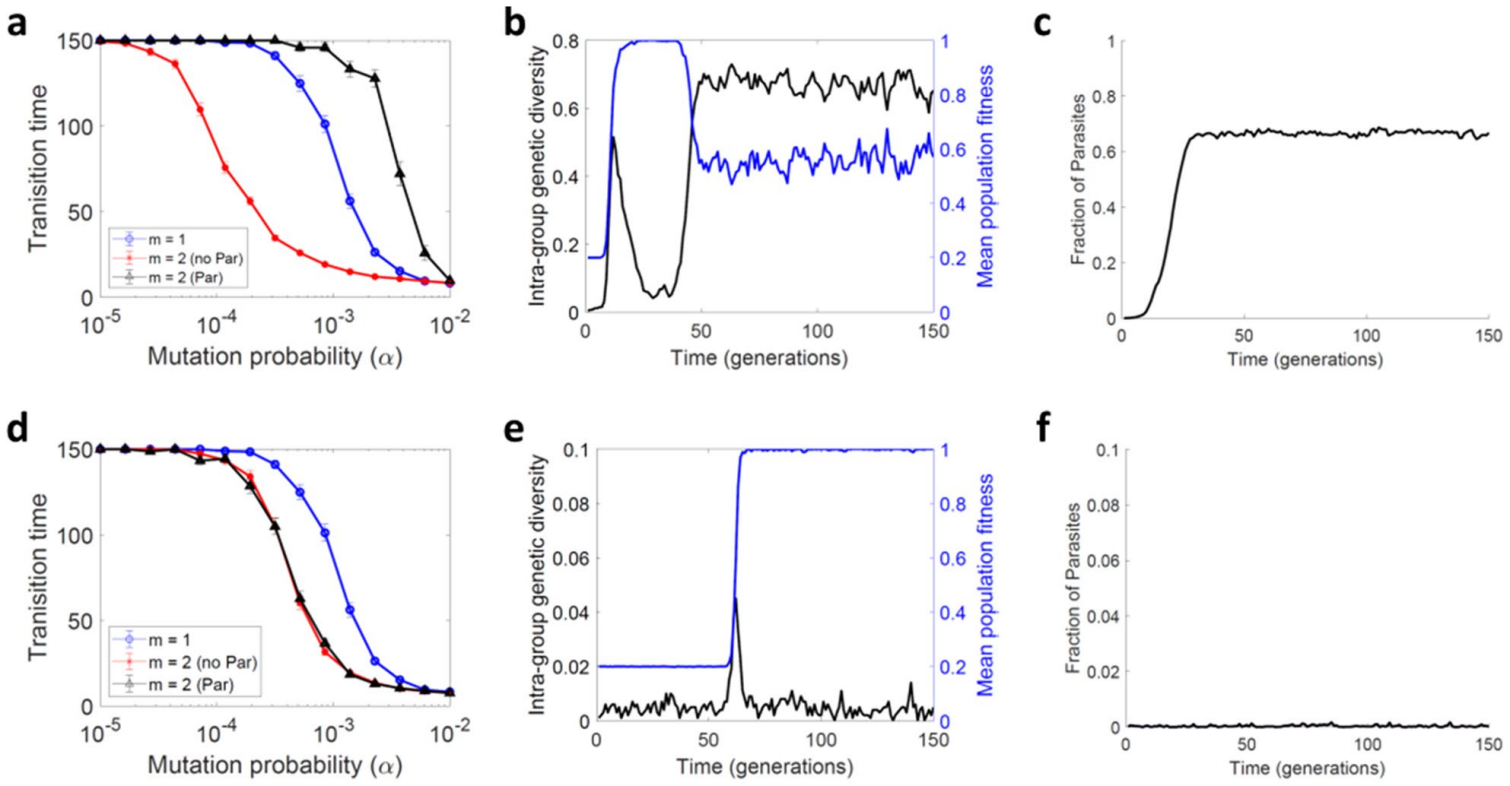
Effect of trans-complementation on evolvability in the presence of genetic parasites. A two-sequence, two-loci, two-allele model was used, allowing for genetic parasites.(**a**–**c)** Random groups. (**d**–**f**) Kin groups (i.e. restricting interactions to sequences derived from a common parental group). (**a**, **d**) Average number of generations required for the population to reach a mean fitness higher than 0.95 as a function of the mutation rate, *α*, for non-interacting sequences (blue, in (**a**) we show the same as in Fig. 2d), groups of helper sequences with no parasites (red, in (**a**) we show the same as in Fig. 2d), and groups of helpers allowing for parasites (black). (**b**, **e**) Association between intra-group genetic diversity (black) and mean population fitness (blue). In each panel, a single simulation is shown to illustrate the association between these two variables. Intra-group genetic diversity (black) was calculated as the fraction of polymorphic alleles within groups, the alleles being “0 helper”, “1 helper” or parasite. (**c**, **f**) Fraction of genetic parasites as a function of time in the same individual simulations. *Parameters* *S* = 10,000, *α*_*C*_ = 0.001, *β*_*C*_ = 5 and in **b**–**c** and **e**–**f**: *α* = 0.001. In **a** and **d** we show average and SEM values obtained from 100 replicate simulations.

We then allowed for genetic parasites, which function as social cheaters. Similar to defective interfering particles in viruses, these were defined as sequences that contained large deletions and hence contributed only partially to complementation (defective), but in addition took a greater share of group-associated fitness to the detriment of helper sequences (interfering). Simulations were started from 0–0 sequences capable of complementing but, in each generation, helper sequences had a certain probability of mutating to parasites. We found that parasites took over the population and reduced the average fitness (Fig. 3a–c). The adverse effect of parasites became gradually stronger as we increased their intra-group selective advantage over helpers, *β*_*C*_ (Figure Supplementary online S1a, c). For *β*_*C*_ > 3, the positive effect of genetic complementation on evolvability was fully cancelled by the presence of parasites since, above this threshold, populations forming groups of two sequences (*m* = 2) no longer adapted faster than those formed by individual sequences (*m* = 1).

To try to avoid parasite invasion, we restricted interactions to sequences that derived from the same group of sequences in the previous generation. We will refer to this type of structure as “kin groups”, as opposed to the randomly formed groups explored above. This describes the way viruses are transmitted using extracellular vesicles or occlusion bodies, as well as direct cell-to-cell viral spread using specialized structures such as viral synapses or plasmodesmata^34^. In all these types of collective spread, group members are viral particles derived from the same producer cell. Unless cells are coinfected with multiple of such groups or free viral particles, the resulting virus-virus interactions are necessarily restricted to progeny derived from a common parental group. We found that, by restricting the interactions to sibling genomes, this type of structures increased genetic relatedness enough to prevent parasite invasion, yet allowed trans-complementation to promote evolvability (Fig. 3d–f). Complementation was evidenced by an increase in intragroup genetic diversity, which was concomitant with an increase in population fitness. This diversity peak was nevertheless transient, since intra-group diversity recovered low values after this episode, leaving fewer opportunities for parasites to take over the population. In contrast, with randomly formed groups, a second wave of increased diversity occurred simultaneously with a reduction in the average fitness of the population due to invasion of the entire population by parasites (Fig. 3b–c).

### Partial trans-complementation evolvability is reduced

In the above simulations, we assumed that genetic defects were fully compensated in trans. Here, we assumed a different situation (average trans-complementation), in which the fitness assigned to a given group (here, a pair) was equal to the average fitness of all allele combinations in the group. For instance, in interactions involving 0–1 and 1–0 sequences, the resulting fitness was equal to the average of fitness values for 0–1, 1–0, 0–0, and 1–1 sequences whereas, in the full trans-complementation model, the resulting fitness was determined solely by the fittest possible combination, that is, the one for 1–1. We found that evolvability was substantially reduced compared to the full trans-complementation case, but that partial complementation still had a positive effect on evolvability (Figure Supplementary S2 online). By analyzing the average fitness effect of mutations, we found that, as opposed to the full complementation case, this system was unable to establish a neutrality scenario that would promote the emergence of the 1–1 sequence.

### Cis-complementation can also foster evolvability

**Figure 4:**
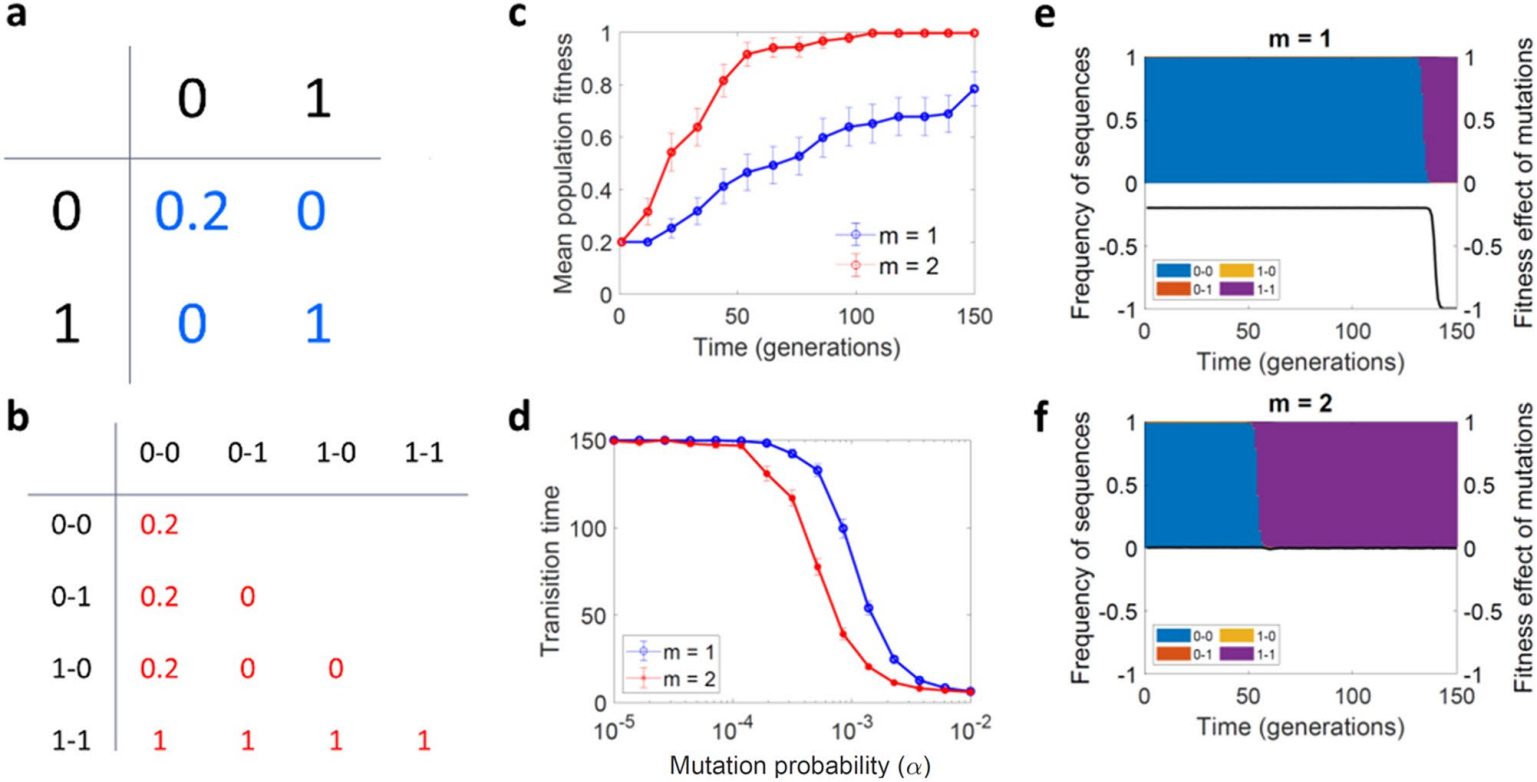
Effects of cis-complementation on evolvability. (**a**) Fitness values for each of the four possible sequences. (**b**) Fitness values for each of the 10 possible two-by-two interactions, calculated as the fitness of the best sequence present. (**c**) Evolution of mean population fitness in the presence (red) or absence (blue) of cis-complementation between pairs of sequences. (**d**) Average number of generations required for the population to reach a mean fitness higher than 0.95, as a function of the mutation rate, *α*. (**e**–**f**) Individual simulations showing the frequency of each sequence (0–0, 0–1, 1–0, and 1–1) and the average fitness effect of individual mutations (black line) as a function of time. *Parameters S* = 10,000, *α*_*C*_ = 0.001 and *β*_*C*_ = 5. and in **c** and **e**–**f**, *α* = 0.001. In **c**–**d** we show the average and SEM from 100 simulations, with the kin-groups transmission method.

Next, we explored a different type of interaction that could be relevant in the context of conformational epistasis. This form of epistasis takes place when one or more sites in the sequence of a protein affect the stability of other sites^9^. Since this concerns variants of a given locus, effects in trans are excluded. Thus, the main difference between this situation and the previous complementation model is that, here, the fitness of the group was a function of the individual performance of each existing sequence, rather than being determined by all combinations of alleles present in different sequences. As above, we allowed for genetic parasites but assumed that populations were structured in kin groups. We first considered a situation in which the fitness value associated with a group was equal to the fitness of the best sequence present in the group (full cis-complementation), and found that under these conditions, evolvability was promoted (Fig. 4). We then considered a model in which the fitness value assigned to a group was equal to the average of the fitness values of each group member (average cis-complementation) and, similar to what we found with trans-complementation, the positive effects on evolvability were reduced, but still observed (Supplementary Figure S3 online).

### Effects of genetic complementation in a multi-locus epistatic model

**Figure 5:**
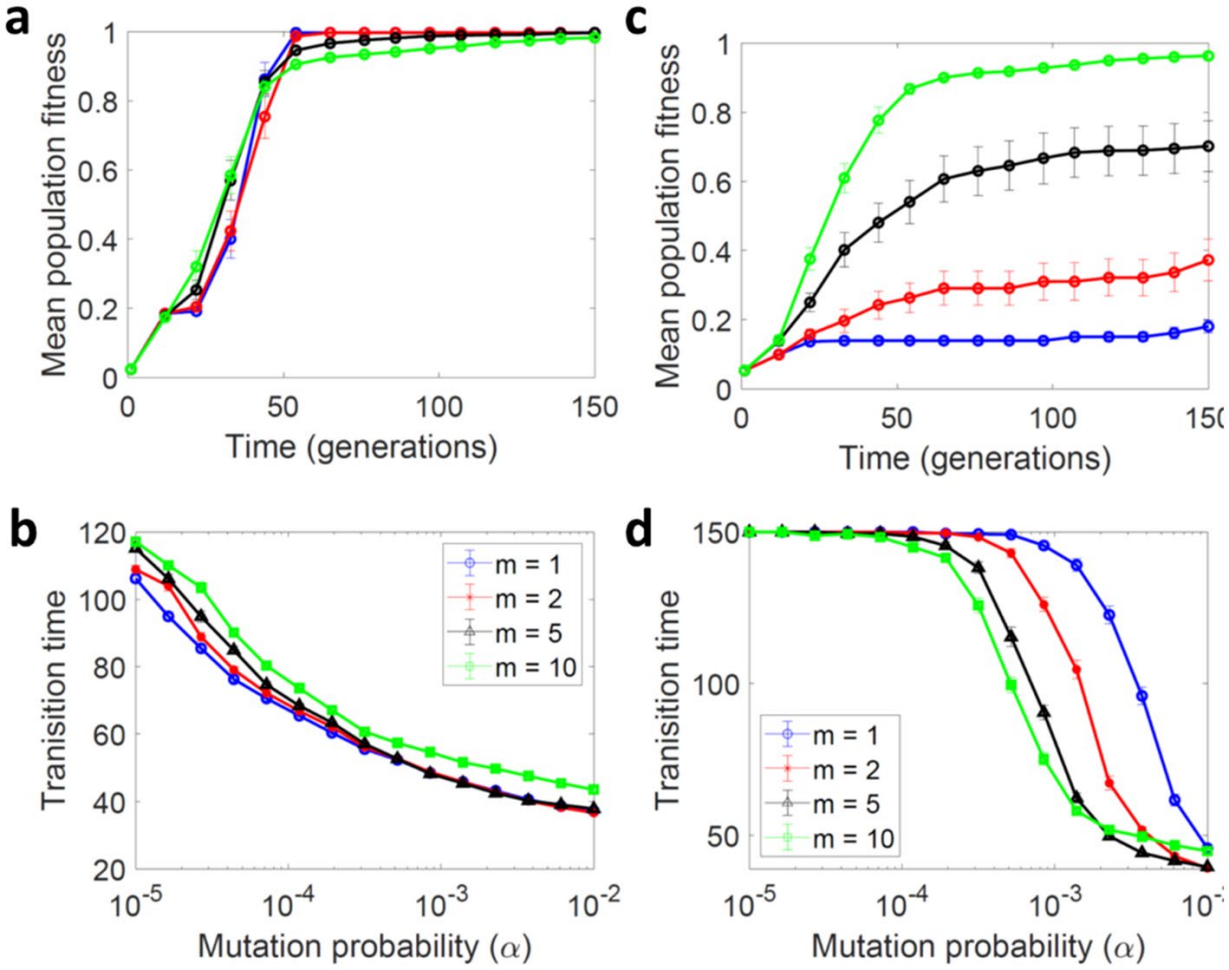
Genetic trans-complementation promotes evolvability in complex fitness landscapes. (**a–b**): Smooth fitness landscape (*K* = 1). (**c**–**d**): Rugged fitness landscape (*K* = 4). (**a**, **c**) Evolution of mean population fitness over time in simulations with non-interacting sequences (blue), and in the presence of full trans-complementation in groups of size *m* = 2 (red), *m* = 5 (black) and *m* = 10 (green). (**b**, **d**) Average number of generations required for the system to reach a mean population fitness higher than 0.95, as a function of the mutation rate, *α*. Genetic parasites were allowed, but populations were structured in kin groups. *Parameters S* = 10,000, *α*_*C*_ = 0.001 and *β*_*C*_ = 5, *α* = 0.001 (in **a** and **c**). The mean and SEM values from 100 simulations are shown.

To generalize our analysis, we used random *NK* models^31^ to create arbitrary fitness landscapes with *N* genes and *K* gene–gene interactions. We also considered groups of *m* > 2 individuals, using the same complementation rules as above. In these simulations, genetic parasites were also allowed but populations were structured in kin groups. We changed *K* (which determines the ruggedness of the fitness landscape) keeping the number of genes constant (*N* = 5). The number of loci that we could consider was subject to computational limitations, since simulation times increased exponentially with *N*. Even if the number of genes in real genomes is typically much higher than five, the actual number of loci determining a given trait and capable of establish epistatic interactions among them should be much smaller than the total number of genes present in a genome. Therefore, *N* = 5 might capture a sufficient level of complexity. As expected, increasing *K* generally hampered evolutionary optimization. With minimal epistasis (*K* = 1), full trans-complementation was slightly detrimental for evolvability (Fig. 5a–b). In contrast, in landscapes with abundant epistasis (*K* = 4), full trans-complementation strongly favored evolvability despite the presence of parasites (Fig. 5c–d). This effect became more pronounced as group size is increased, since mutual compensation of genetic defects was more likely in larger groups. However, this positive effect on evolvability was reversed when trans-complementation was only partial (average trans-complementation; Supplementary Figure S4 online).

We also applied the *NK* model to the cis-complementation case. As above, we allowed for parasites but restricted interactions to progeny derived from the same parental group. We found that evolvability was also fostered by this type of complementation, but that this effect strongly depended on whether group-associated fitness was determined by the fittest sequence (full cis-complementation; Figure S5 online) or by the average fitness value of all group members (average cis-complementation; Supplementary Figure S6 online). The results were qualitatively similar to those obtained with the trans-complementation model, since evolvability was fostered under full complementation, but not when complementation was partial. We conclude that the main benefit of sequence interactions does not reside in the ability to explore novel allele combinations, as this was not possible in the cis-complementation case. Instead, complementation promotes evolvability by allowing low-fitness sequences to be maintained in the population, thereby increasing the ability of the population to traverse rugged fitness landscapes.

### Complementation fosters exploration of rugged fitness landscapes

**Figure 6:**
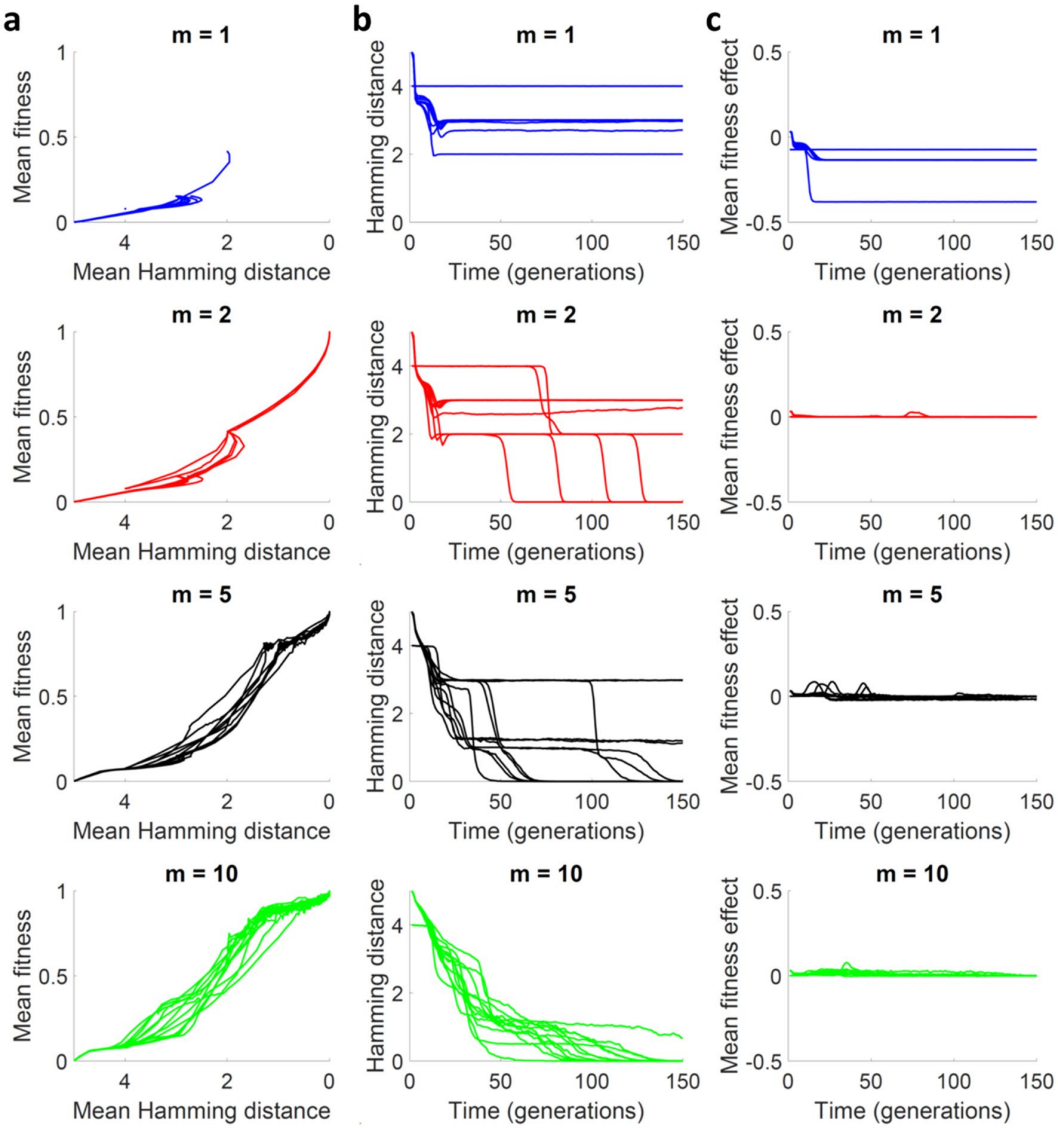
Trans-complementation fosters exploration of complex fitness landscapes. (**a**) Mean population fitness as a function of the mean Hamming distance of the population to the optimum over 10 individual simulations. (**b**) Average Hamming distance of the population to the optimum, as a function of time. (**c**) Average fitness effect of mutations as a function of time. For each type of plot, the four graphs show the results obtained with four different *m*-values, as indicated. A full trans-complementation model with parasites and kin groups was used. *Parameters* *N* = 5, *K* = 4, *S* = 10,000, *α* = 0.0005, *α*_*C*_ = 0.001 and *β*_*C*_ = 5.

To better understand how complementation promoted evolvability, we inspected evolutionary trajectories in the rugged fitness landscape (*N* = 5, *K* = 4). As above, in these simulations we allowed for parasites, but populations were structured in kin groups. We plotted mean population fitness along the 150-generation simulations as a function of the mean Hamming distance of the population to the highest-fitness sequence (global optimum), starting each simulation from a different position in the landscape. In the absence of interactions (*m* = 1), populations were systematically trapped in local sub-optimal peaks. In contrast, when full trans-complementation was allowed, populations reached maximal fitness. This positive effect was stronger as group size, *m*, increased (Fig. 6a–b). This is appreciated when analyzing the trajectories of the system through the genotype–phenotype map. With null or low complementation (*m* = 1 or *m* = 2), the adaption process is restricted to the path allowed by additive mutations, which usually traps the system in local maxima. With abundant complementation (*m* = 5 or *m* = 10), new evolutionary trajectories emerge, since now the system is able to accumulate low fitness sequences. The analysis of the average fitness effect (the same way as in the cases of the simple system), confirmed that, in populations displaying complementation, mutations had much smaller fitness effects than in populations of non-interacting sequences, as expected in a relaxed-selection scenario (Fig. 6c). We repeated the analysis with the full cis-complementation model, and it can be seen in Figure S7 online that the phenomenon is the same, with just simple quantitative changes, which is not surprising, since we have already found that cis-complementation exhibits reduced evolvability versus trans-complementation.

## Discussion

We have shown through simulations that a form of cooperation based on compensation of genetic defects promotes adaptability by enhancing the capacity of the population to explore complex fitness landscapes. While the population and genetic factors that determine the evolutionary stability of cooperation have been extensively investigated^35–44^, how cooperation feeds back on evolvability is a less explored topic. Moreover, many previous studies have analyzed how populations evolve in complex fitness landscapes^1–3,10–14^, but these have not addressed how cooperation among genetically distinct individuals determines evolvability. Only in^45^ is this connection explored, where the authors determine that cooperation by public goods enhances adaptability by promoting peak shifts in complex fitness landscapes. Our results continue this line, showing a clear connection between cooperation and adaptability.

A fundamental question in evolutionary biology is how cooperation can be favored by natural selection despite the costs associated with helping others. This conundrum has been addressed by different approaches, introducing kin and group selection, and solved using frequency-dependent selection models and game theory, among other approaches^35–42^. In general, for cooperation to be evolutionarily stable, cheaters need to be segregated from cooperative interactions. In the absence of such assortment, cheaters are favored by selection because they reap the benefits of cooperation but do not pay the costs of helping others. Cheater invasion leads to a reduction of mean population fitness and, potentially, to loss of the cooperative trait. Both kin and group selection models show that cheater invasion can be avoided when interactions occur preferentially between genetically related individuals. Not surprisingly, we also found that genetic complementation was severely jeopardized by genetic parasites, and that restricting interactions to groups of genealogically related sequences could prevent parasite invasion. Intuitively, it may be expected that, by increasing relatedness, such assortment should also curtail the benefits of genetic complementation for adaptation in rugged landscapes. However, interestingly, our model shows that there is room to avoid parasites while still promoting evolvability.

In our simulations, we have referred to individual sequences that establish pairwise or higher-order interactions involving different types of genetic complementation. As discussed, the model could be applied to highly diverse RNA virus populations exhibiting frequent coinfections. Recent work has shown that viruses often undergo inter-host transmission and intra-host dissemination as groups of viral particles^21^, increasing coinfection probability and, thus, the opportunities for genetic complementation. This possibility was suggested verbally in previous work^46,47^, but the effects of complementation on evolvability were not explored by simulations, models, or experiments, and the problem of genetic parasites was not addressed.

Although we have discussed our model in the context of viral evolution, there may be other biological systems that exhibit similar interactions. For instance, by sharing gene products encoded on different chromosomes, sexually-reproducing diploid or polyploid organisms tend to buffer the effects of maladaptive alleles, as evidenced by the fact that most strongly deleterious alleles are recessive^48–50^. The potential evolutionary advantages and disadvantages of diploids over haploids have been investigated^51,52^, including with respect to evolvability. Genetic algorithm models have pointed out that sexual reproduction in diploid organisms can accelerate optimization due to recombination, which allows for a more rapid emergence of novel allelic combinations^53,54^. However, to our knowledge, previous work has not considered genetic complementation as a process that increases the evolvability of diploids relative to haploids. On the other hand, the role of gene or genome duplications in promoting long-term evolvability has been addressed previously. Provided that the original gene function is conserved in at least one copy, duplicated genes can accumulate cryptic variation that can lead to the evolution of novel functions in the long term^55–57^. It has also been pointed out that duplicated genes can be preserved if different copies undergo mutually compensable deleterious mutations^58^. However, as we have shown, increased ploidy may not per se foster evolvability unless selfish DNA elements are prevented.

Genetic complementation has also been suggested to play a role in evolutionary transitions such as from unsegmented to segmented viruses^59^, and in the zoonotic transmission of influenza A virus^60^. Furthermore, complementation could be relevant to the evolution of symbiotic relationships in which certain metabolic pathways become redundant^61^, allowing one or both partners to evolve new functions. Finally, in complex social species, compensation of deleterious mutations through community-level interactions might allow the accumulation of genetic diversity that could yield new adaptations.

## Supplementary Information

Supplementary Information.

## Data Availability

All data showed are generated from simulations with the models described in the Methods section. Scripts are available upon request to Ernesto.Segredo@uv.es.
