# Supplementary material for: Genetic complementation fosters evolvability in complex fitness landscapes
Source: Sci Rep. 2023 Jan 12;13:662. doi: 10.1038/s41598-022-26588-y (PMC9837146; doi:10.1038/s41598-022-26588-y)
Supplement: Supplementary file 1 — Supplementary Information. [file 41598_2022_26588_MOESM1_ESM.pdf]

Genetic complementation fosters evolvability in complex fitness landscapes

Ernesto Segredo-Otero and Rafael Sanjuán*

Institute for Integrative Systems Biology (I2SysBio), Consejo Superior de Investigaciones Científicas-Universitat de València, C/ Catedrático Agustín Escardino 9, 46980 Paterna, València, Spain

*Correspondence: rafael.sanjuan@uv.es

Supplementary material

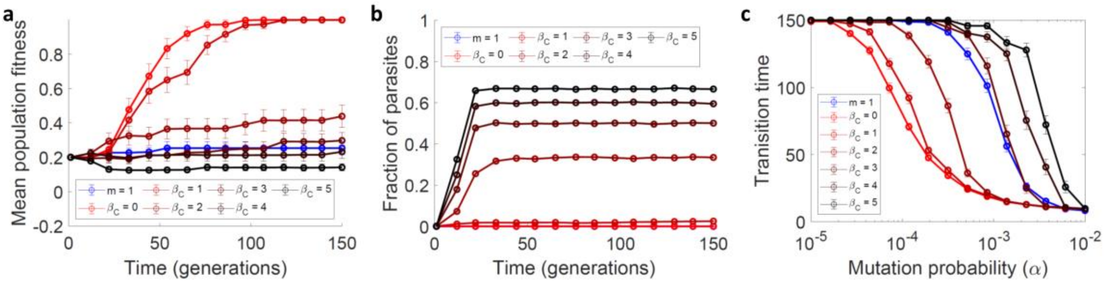

Figure S1. Effect of the intra-group competitive advantage of parasites on the ability of genetic complementation to promote evolvability. A two-sequence, two-locus, two-allele model was considered. Sequence pairs were established randomly. **A.** Evolution of mean population fitness. **B**. Evolution of the fraction of parasites. **C.** Average number of generations required for the system to reach a mean population fitness higher than 0.95, as a function of the mutation rate, α. Simulations were performed in the absence (blue) or in the presence of genetic complementation with m = 2. In the later case, shades of red to black are used to indicate parasites with different levels of intra-group competitive advantage (from β_C = 0 to β_C = 5). Parameters: S = 10,000, α_C = 0.001, and in A-B, α = 0.001.

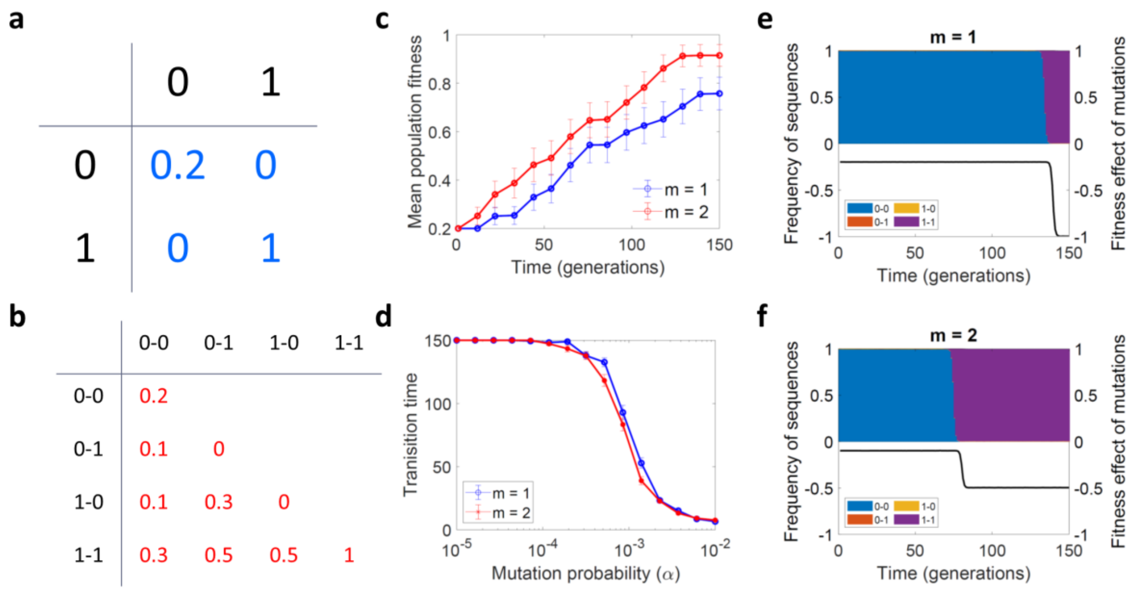

Figure S2. Effects of partial trans-complementation on evolvability. A two-sequence, two-locus, two-allele model was considered. Genetic parasites were allowed and sequence pairs were established using the kin groups model. **A.** Fitness values for each of the four possible sequences. **B.** The fitness values of each of the 10 possible two-by-two interactions were calculated by averaging the fitness values of all possible allele combinations. **C.** Evolution of mean population fitness in the presence (red) or absence (blue) of trans-complementation between pairs of sequences. **D.** Average number of generations required for the population to reach a mean fitness higher than 0.95 as a function of the mutation rate, α. **E-F.** Individual simulations showing the frequency of each sequence (0-0, 0-1, 1-0, and 1-1) and the average fitness effect of individual mutations (black line) as a function of time. Parameters: S = 10,000, α_C = 0.001, β_C = 5. and in C and E-F, α = 0.001. In C-D we show the average and SEM from 100 simulations.

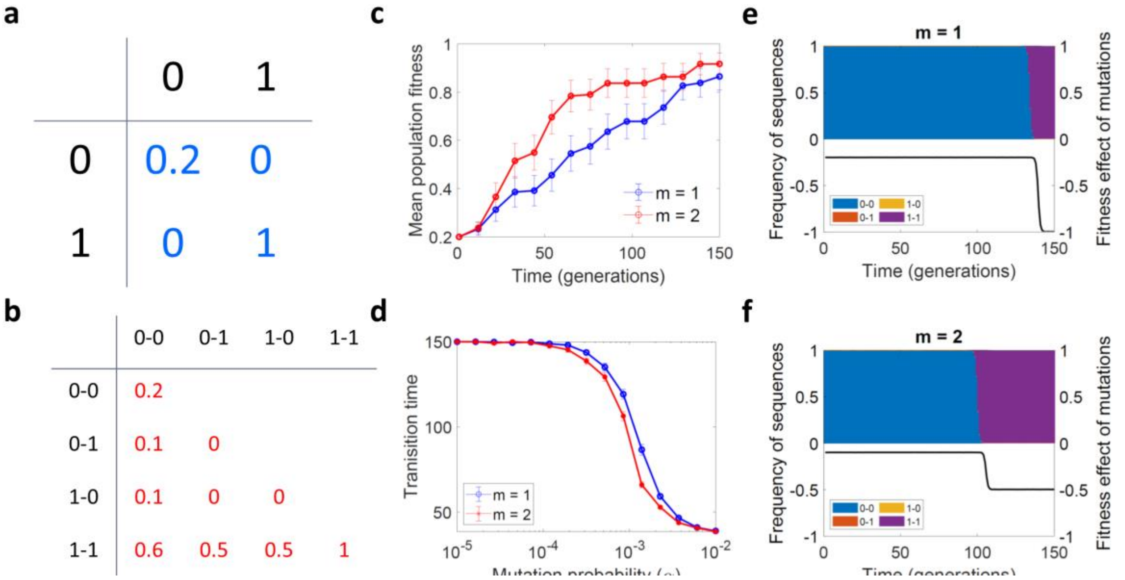

Figure S3. Effects of partial cis-complementation on evolvability. A two-sequence, two-locus, two-allele model was considered. Genetic parasites were allowed and sequence pairs were established using the kin groups model. **A.** Fitness values for each of the four possible sequences. **B.** Fitness values for each of the 10 possible two-by-two interactions, which was calculated as the average of fitness values for each sequence present. **C.** Evolution of mean population fitness in the presence (red) or absence (blue) of trans-complementation between pairs of sequences. **D.** Average number of generations required for the population to reach a mean fitness higher than 0.95 as a function of the mutation rate, α. **E-F.** Individual simulations showing the frequency of each sequence (0-0, 0-1, 1-0, and 1-1) and the average fitness effect of individual mutations (black line) as a function of time. Parameters: S = 10,000, α_C = 0.001, β_C = 5. and in C and E-F, α = 0.001. In C-D we show the average and SEM from 100 simulations.

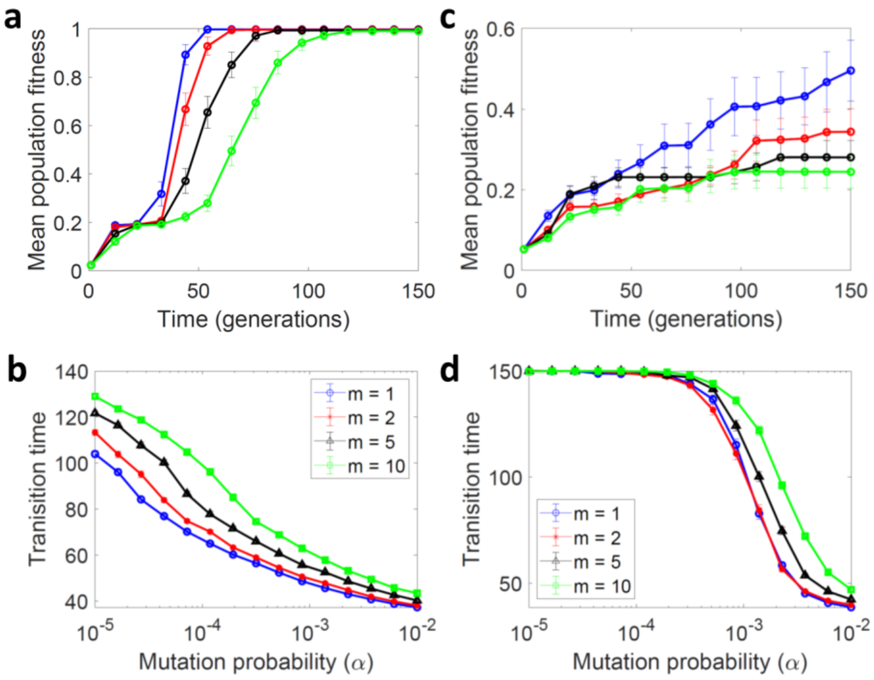

Figure S4. Partial trans-complementation does not foster evolvability in complex fitness landscapes. Five loci were considered, genetic parasites were allowed, and sequence groups were established using the kin groups model. **A-B:** Smooth fitness landscape ($K = 1$). **C-D**: Rugged fitness landscape (K = 4). **A, C**. Evolution of mean population fitness over time in simulations with non-interacting sequences (blue), and interactions in groups of size m = 2 (red), m = 5 (black) and m = 10 (green). **B, D.** Average number of generations required for the population to reach a mean fitness higher than 0.95, as a function of the mutation rate, α. Parasites were allowed and populations were structured in kin groups. Parameters: S = 10,000, α_C = 0.001 and β_C = 5, and in A-C, α = 0.001. The mean and SEM values from 100 simulations are shown.

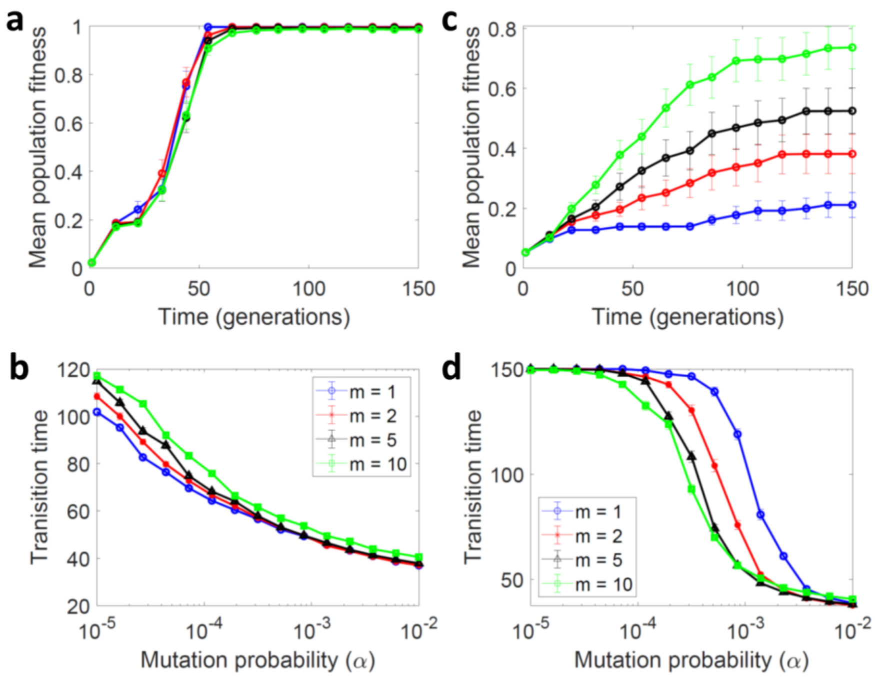

Figure S5. Full cis-complementation fosters evolvability in complex fitness landscapes. Five loci were considered, genetic parasites were allowed, and sequence groups were established using the kin groups model. **A-B:** Smooth fitness landscape (K = 1). **C-D**: Rugged fitness landscape (K = 5). **A, C**. Evolution of mean population fitness over time in simulations with non-interacting sequences (blue), and in the presence of full trans-complementation in groups of size m = 2 (red), m = 5 (black) and m = 10 (green). **B, D.** Average number of generations required for the population to reach a mean fitness higher than 0.95, as a function of the mutation rate, α. Parameters: S = 10,000, α_C = 0.001 and β_C = 5, and in A-C, α = 0.001. The mean and SEM values from 100 simulations are shown.

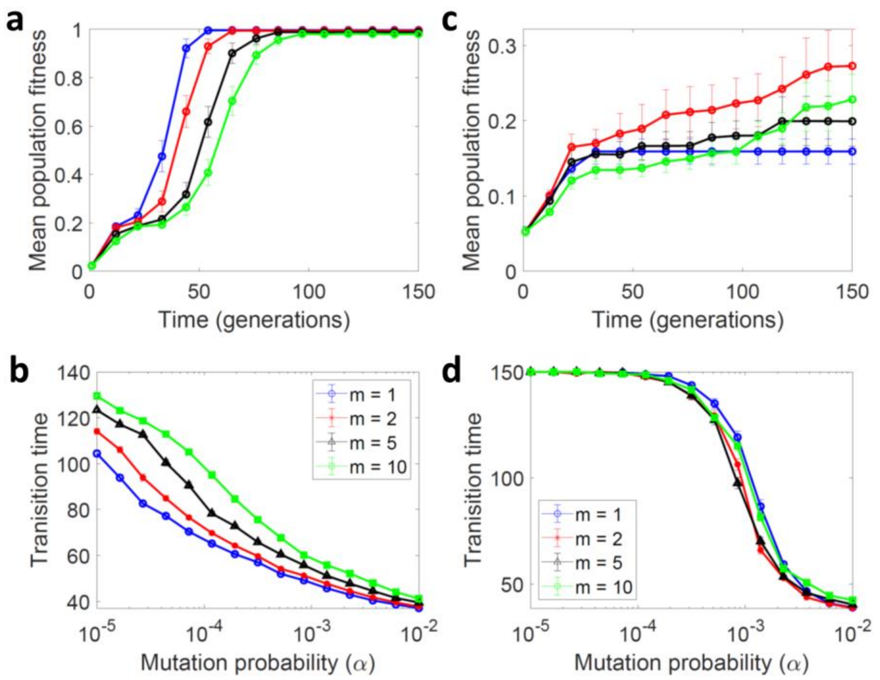

Figure S6. Average cis-complementation shows little effect on evolvability in complex fitness landscapes. Five loci were considered, genetic parasites were allowed, and sequence groups were established using the kin groups model. **A-B:** Smooth fitness landscape (K = 1). **C-D**: Rugged fitness landscape (K = 5). **A, C**. Evolution of mean population fitness over time in simulations with non-interacting sequences (blue), and in the presence of full trans-complementation in groups of size m = 2 (red), m = 5 (black) and m = 10 (green). **B, D.** Average number of generations required for the population to reach a mean fitness higher than 0.95, as a function of the mutation rate, α. Parameters: S = 10,000, α_C = 0.001 and β_C = 5, and in A-C, α = 0.001. The mean and SEM values from 100 simulations are shown.

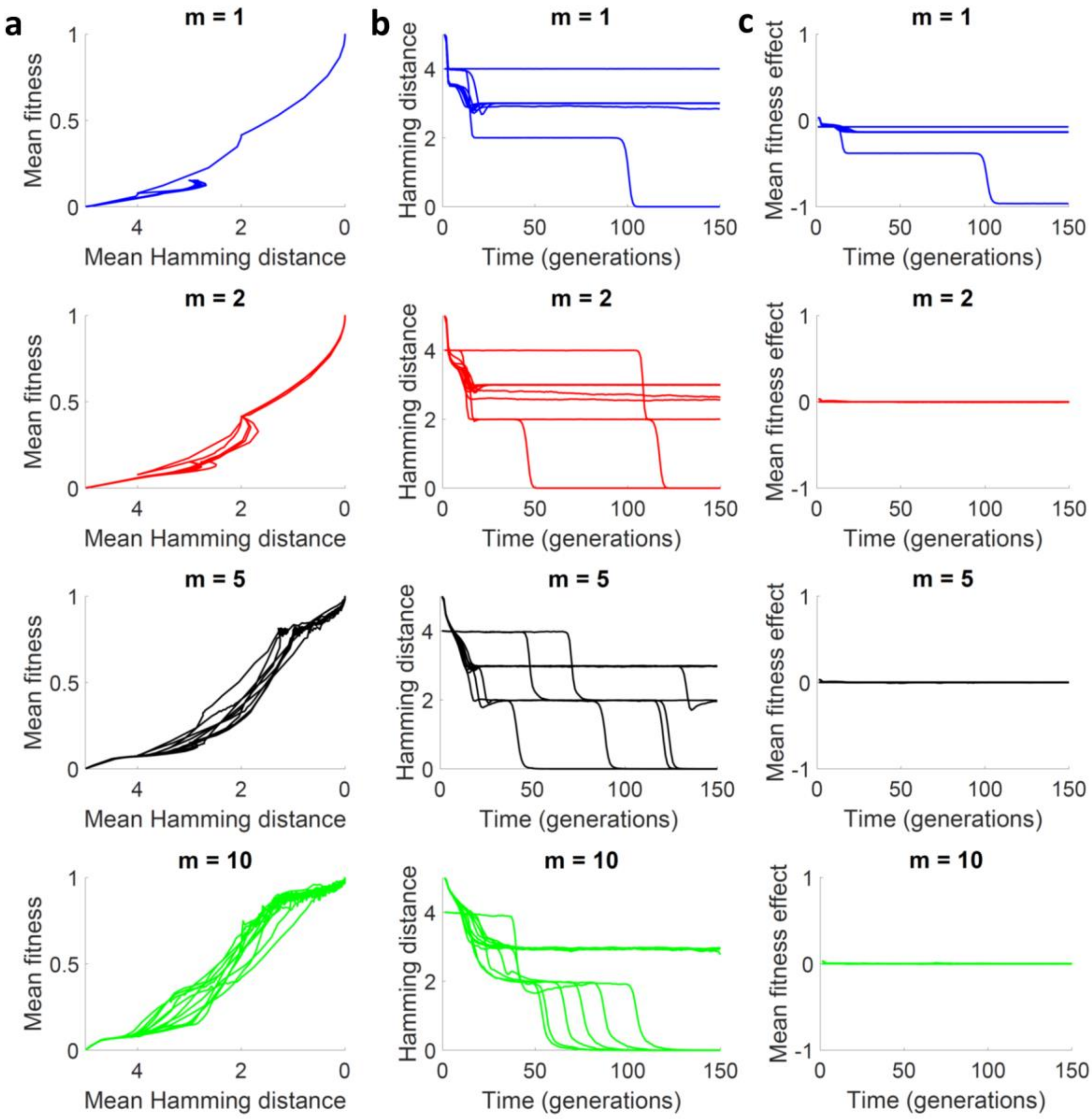

Figure S7. Cis-complementation fosters exploration of complex fitness landscapes. A. Mean population fitness as a function of the mean Hamming distance of the population to the optimum over 10 individual simulations. **B.** Average Hamming distance of the population to the optimum, as a function of time. **C.** Average fitness effect of mutations as a function of time. For each type of plot, the four graphs show the results obtained with four different m-values, as indicated. A full trans-complementation model with parasites and kin groups was used. Parameters: N = 5, K = 4, S = 10,000, α = 0.0005, α_C = 0.001 and β_C = 5.
